# Age-stratified heritability estimation in the Framingham Heart Study families

**DOI:** 10.1186/1471-2156-4-S1-S32

**Published:** 2003-12-31

**Authors:** W Mark Brown, Stephanie R Beck, Ethan M Lange, Cralen C Davis, Christine M Kay, Carl D Langefeld, Stephen S Rich

**Affiliations:** 1 Department of Public Health Sciences, Wake Forest University School of Medicine, Winston-Salem, North Carolina, USA

## Abstract

The Framingham Heart Study provides a unique source of longitudinal family data related to CVD risk factors. Age-stratified heritability estimates were obtained over three age groups (31–49 years, 50–60 years, and 61–79 years), reflecting the longitudinal nature of the data, for four quantitative traits. Age-adjusted heritability estimates were obtained at a single common time point for the same four quantitative traits. The importance of these groups is that they consist of the same individuals. The highest age-stratified heritability estimate (h^2 ^= 0.88 (± 0.06)) was for height in the model adjusting for gender over all three age groups. SBP gave the lowest heritability estimate (h^2 ^= 0.15 (± 0.11)) for the 70 age group in the model adjusting for gender, height, BMI, smoker, and drinker. BMI had slightly higher estimates (h^2 ^= 0.64 (± 0.11)) in the 40 age group than previously published. The highest age-adjusted heritability estimate (h^2 ^= 0.90 (± 0.06)) was for height in the model adjusting for gender. SBP gave the lowest heritability estimate (h^2 ^= 0.38 (± 0.09)) for unadjusted model. These results indicate that some common, complex traits may vary little in their genetic architecture over time and suggest that a common set of genes may be contributing to observed variation for these longitudinally collected phenotypes.

## Background

Cardiovascular disease (CVD) has a complex genetic basis. There are major risk factors that cannot be changed-heredity, gender, and increasing age. Many risk factors can be changed — obesity, high blood pressure, smoking, high cholesterol levels, physical inactivity, stress, and substance abuse. Many of these modifiable risk factors have a genetic basis (obesity, blood pressure, total cholesterol) or, at a minimum, tend to aggregate in families (smoking, personality traits). These factors also change over time in prevalence and potential effect on phenotype (an age-specific penetrance). By examining age-stratified heritability associated with common risk factors, a better understanding of the genetic contribution to their phenotypic variance can be made. In addition, estimation of the heritability of these factors as surrogates of age-specific penetrance can be used to test constancy across age groups.

One approach in better understanding the genetic basis of CVD is to study the genetic basis of the underlying quantitative traits. There are several advantages for studying quantitative trait phenotypes. These advantages include 1) information from all family members can be used in the analysis, not just those who are considered "affected", and 2) the strength of genetic control of these phenotypic determinants (heritability) may be substantial and, therefore, more amenable to genetic mapping than the qualitative trait. Narrow-sense heritability (h^2^) for selected quantitative trait phenotypes [height, weight, body mass index (BMI) and systolic blood pressure (SBP)] will be estimated using a variance component approach [1] in the Framingham Heart Study (FHS), a longitudinal study of CVD risk factors.

## Methods

The FHS is a longitudinal, community-based study that also included spouses and offspring of the original FHS cohort. Data was provided on 330 pedigrees from FHS consisting of 4692 subjects, in which 2885 had data and some genetic information. Three age groups were partitioned out at time points 40 (± 9), 55 (± 5), and 70 (± 9) years. In order to provide a constant cohort size for data analysis, each subject had to have a "key" phenotype measured for each time period.

The value associated for each phenotype was taken at the closest available time point to the three age classes. These three age groups were chosen to give a broad timeline for comparison. Given that the majority of the data seemed to center around the middle age group, we broadened the outer ranges in an effort to keep a wide enough time frame between the three different groups. First, the closest time point around age 55 was selected. Next, data for age groups 40 and 70 were chosen. Given data at the middle of the 55 age group, the data point for the age 40 group would have be chosen as close to 40 as possible. If a participant did not have data at age 40, then our attempt was to take an earlier time point rather than a later one to try and keep the time groups broad. The same was true for the age 70 group. We wanted to avoid having data points for an individual for time points like 49, 55, and 61. We tried to maintain a minimum of 15 years between each of the three age groups. If a participant did not have a value for the three time points, then that individual entered the analysis with missing data for all three age classes. Height was considered constant and if a time point had a missing height, the value that preceded the missing time point was used. BMI was calculated for only those times that contained weight. Weight, BMI, and SBP were all log-transformed as dependent variables (traits), which better approximated the distributional assumptions. Untransformed values for weight, BMI, and SBP were used when entered as covariates. For age-adjusted analysis, time periods were aligned to be consistent across the cohorts. Age was not used as a covariate in the age-stratified analyses because age was the stratifying variable. The corresponding year 12 from entry time point was used (time point 7 in Cohort 1; time point 3 in Cohort 2). This time point represented the majority of the participants (781 out of the 795) used in the stratified analyses. By maintaining a consistent sample group, comparison between the two (age-stratified and age-adjusted) analyses are more applicable.

Heritability (h^2^) estimates were determined using Sequential Oligogenic Linkage Analysis Routines (SOLAR) [2]. A family was included in the h^2 ^estimates if it contained at least one sib pair or one avuncular pair. Significance of the estimated heritability was determined by likelihood ratio tests, in which the obtained likelihood of the model with the additive genetic variance component and covariates was compared with the obtained likelihood of the model with the additive genetic variance component, constrained to be zero. Relationship pair counts were performed using Statistical Analysis for Genetic Epidemiology (S.A.G.E.) [3].

## Results

### BMI

BMI (log-transformed) had the highest residual heritability (h^2 ^= 0.64 ( ± 0.11)) for the model with gender, smoker, and drinker as covariates in the 40-year age group (Table 1). The lowest estimated residual heritability for BMI was h^2 ^= 0.42 ( ± 0.09) for the model containing gender as the only covariate in the 55-year age group. The age-adjusted analysis performed at year 12 had the highest residual heritability of h^2 ^= 0.53 ( ± 0.10) in the model containing gender and height as covariates. The lowest estimated residual heritability for BMI was h^2 ^= 0.46 ( ± 0.10) for the model containing gender as the only covariate.

### Weight

Weight (log-transformed) had the highest residual heritability (h^2 ^= 0.63 (± 0.09)) for the model with gender, height, smoker, and drinker as covariates in the 40-year age group (Table 2). The lowest estimated residual heritability for weight was h^2 ^= 0.44 (± 0.10) for the unadjusted model in the 55-year age group. The age-adjusted analysis performed at year 12 had the highest residual heritability of h^2 ^= 0.52 (± 0.10) in the model containing gender, height, and smoker as covariates. The lowest estimated residual heritability for weight was h^2 ^= 0.42 (± 0.10) for the model containing age as the only covariate.

### Height

Height (untransformed) had the highest residual heritability (h^2 ^= 0.88 (± 0.06)) for the model with gender as the only covariate in all three age groups (Table 3). The lowest estimated residual heritability for height was h^2 ^= 0.48 (± 0.09) for the unadjusted model in the 70-year age group. The age-adjusted analysis performed at year 12 had the highest residual heritability of h^2 ^= 0.90 (± 0.06) in the model containing gender as a covariate. The lowest estimated residual heritability for height was h^2 ^= 0.52 (± 0.09) for the model containing age as the only covariate.

### Systolic Blood Pressure (SBP)

SBP (log-transformed) had the highest residual heritability (h^2 ^= 0.39 (± 0.11)) for the model with gender, BMI, smoker, and drinker as covariates in the 40-year age group (Table 4). The lowest estimated residual heritability for SBP was h^2 ^= 0.17 (± 0.09) for the unadjusted model in the 55-year age group. The age-adjusted analysis performed at year 12 had the highest residual heritability of h^2 ^= 0.47 (± 0.11) in the model containing gender, BMI, and drinker as covariates. The lowest estimated residual heritability for SBP was h^2 ^= 0.38 (± 0.09) for the model containing age as the covariate.

All models that estimated heritability for BMI, weight, and height were highly significant for rejecting the null hypothesis of h^2 ^= 0, with *p*-values < 0.0000025. For SBP, 8 of the 20 models strongly suggested that the heritability of SBP was highly significantly different from zero (*p *< 0.001), with no models not reaching significance (*p *< 0.034).

## Discussion

A long-standing concept in animal and plant genetics is that, over time, the relative contribution of genes to a phenotype decreases. This decrease may be due, in part, to the accumulation of environmental insults that tends to increase the total phenotypic variance while maintaining a constant (additive) genetic variance, resulting in lower heritability estimates over time. Alternatively, different sets of genes could be contributing to the variance of a phenotype over time, resulting in an unpredictable (but not always decreasing) change in heritability. As this concept has not been thoroughly examined in humans, the data from the FHS represents an opportunity to test these hypotheses.

In this application of variance component methods, a decision was made to enhance the comparability of analyses from different age groups by requiring a participant to have the phenotypic value in all three age groups. In this fashion, 795 participants in 170 families were included in the analyses (Table 5). The resulting family structure revealed that almost all families were nuclear with at least one sibling pair. The reduction in sample size and complexity caused low power to detect even the modest LOD scores for lower heritability estimates. Of the four traits analyzed at the three specific age groups, three exhibited high heritability estimates (>0.60) for some model — BMI (h^2 ^= 0.64 (± 0.11)), weight (h^2 ^= 0.63 (± 0.11)), and height (h^2 ^= 0.88 (± 0.06)). These estimates are somewhat higher than reported in previous studies [4,5]. Of these three, only height showed an increase using the year 12 analysis (h^2 ^= 0.90 (± 0.06)). The year 12 analysis for BMI and weight fell within the range presented across the three age groups. SBP showed a larger heritability estimate (h^2 ^= 0.47 (± 0.11)) in the year 12 analysis than the age-stratified analysis (h^2 ^= 0.39 (± 0.11)) and was closer to the maximum values in other studies. Overall, the heritability estimates seemed consistent over the age groups and with the year 12 age-adjusted group because the estimates were within one standard error of each other with almost all models. Based on these results, it is still unclear whether doing age-stratified analysis or age-adjusted analysis fits longitudinal data in a preferred method.

## Figures and Tables

**Table 1:** Heritability estimates for log transformed BMI

	Trait: BMI (log)		
	
Covariates	h^2^r ± SE	*p*-value	variance due to covariates (%)
Unadjusted			
Age Group 40	0.53 ± 0.09	<0.0000001	-
Age Group 55	0.43 ± 0.09	<0.0000006	-
Age Group 70	0.43 ± 0.09	<0.0000003	-
Year 12 (age adjusted)	0.46 ± 0.10	<0.0000007	2
Gender			
Age Group 40	0.53 ± 0.09	<0.0000001	2
Age Group 55	0.42 ± 0.09	<0.0000007	2
Age Group 70	0.43 ± 0.09	<0.0000003	0.1
Year 12 (age adjusted)	0.46 ± 0.10	<0.0000004	4
Gender, smoker			
Age Group 40	0.60 ± 0.09	<0.0000001	5
Age Group 55	0.51 ± 0.09	<0.0000001	3
Age Group 70	0.51 ± 0.09	<0.0000001	3
Year 12 (age adjusted)	0.53 ± 0.10	<0.0000001	6
Gender, drinker			
Age Group 40	0.59 ± 0.11	<0.0000001	3
Age Group 55	0.50 ± 0.11	<0.0000010	2
Age Group 70	0.48 ± 0.11	<0.0000023	0.3
Year 12 (age adjusted)	0.50 ± 0.13	<0.0000204	5
Gender, smoker, drinker			
Age Group 40	0.64 ± 0.11	<0.0000001	5
Age Group 55	0.55 ± 0.11	<0.0000001	3
Age Group 70	0.53 ± 0.11	<0.0000002	3
Year 12 (age adjusted)	0.52 ± 0.12	<0.0000035	7

**Table 2:** Heritability estimates for log transformed weight

	Trait: Weight (log)		
	
Covariates	h^2^r ± SE	*p*-value	variance due to covariates (%)
Unadjusted			
Age Group 40	0.51 ± 0.09	<0.0000001	-
Age Group 55	0.44 ± 0.10	<0.0000009	-
Age Group 70	0.45 ± 0.09	<0.0000001	-
Year 12 (age adjusted)	0.42 ± 0.10	<0.0000051	1
Gender, height			
Age Group 40	0.60 ± 0.09	<0.0000001	46
Age Group 55	0.51 ± 0.09	<0.0000001	41
Age Group 70	0.51 ± 0.09	<0.0000001	35
Year 12 (age adjusted)	0.45 ± 0.10	<0.0000008	43
Gender, height, smoker			
Age Group 40	0.58 ± 0.11	<0.0000001	44
Age Group 55	0.49 ± 0.11	<0.0000021	40
Age Group 70	0.49 ± 0.11	<0.0000025	33
Year 12 (age adjusted)	0.52 ± 0.10	<0.0000001	44
Gender, height, drinker			
Age Group 40	0.63 ± 0.11	<0.0000001	45
Age Group 55	0.53 ± 0.11	<0.0000001	41
Age Group 70	0.52 ± 0.11	<0.0000003	36
Year 12 (age adjusted)	0.48 ± 0.12	<0.0000334	42
Gender, height, smoker, drinker			
Age Group 40	0.63 ± 0.09	<0.0000001	31
Age Group 55	0.53 ± 0.09	<0.0000001	29
Age Group 70	0.51 ± 0.09	<0.0000001	21
Year 12 (age adjusted)	0.51 ± 0.12	<0.0000057	44

**Table 3:** Heritability estimates for height (untransformed)

	Trait: Height		
	
Covariates	h^2^r ± SE	*p*-value	variance due to covariates (%)
Unadjusted			
Age Group 40	0.53 ± 0.09	<0.0000001	-
Age Group 55	0.55 ± 0.09	<0.0000001	-
Age Group 70	0.48 ± 0.09	<0.0000001	-
Year 12 (age adjusted)	0.52 ± 0.09	<0.0000001	<0.1
Gender			
Age Group 40	0.88 ± 0.06	<0.0000001	53
Age Group 55	0.88 ± 0.06	<0.0000001	52
Age Group 70	0.88 ± 0.06	<0.0000001	53
Year 12 (age adjusted)	0.90 ± 0.06	<0.0000001	53

**Table 4:** Heritability estimates for log transformed SBP

	Trait: SBP (log)		
	
Covariates	h^2^r ± SE	*p*-value	variance due to covariates (%)
Unadjusted			
Age Group 40	0.27 ± 0.09	<0.0014716	-
Age Group 55	0.17 ± 0.09	<0.0177473	-
Age Group 70	0.21 ± 0.09	<0.0044043	-
Year 12 (age adjusted)	0.38 ± 0.09	<0.0000094	3
Gender, BMI			
Age Group 40	0.33 ± 0.10	<0.0001899	13
Age Group 55	0.24 ± 0.09	<0.0035443	12
Age Group 70	0.22 ± 0.09	<0.0033321	4
Year 12 (age adjusted)	0.40 ± 0.09	<0.0000064	9
Gender, BMI, smoker			
Age Group 40	0.31 ± 0.10	<0.0010109	15
Age Group 55	0.23 ± 0.10	<0.0059096	11
Age Group 70	0.24 ± 0.09	<0.0024420	4
Year 12 (age adjusted)	0.40 ± 0.10	<0.0000116	7
Gender, BMI, drinker			
Age Group 40	0.39 ± 0.11	<0.0002247	15
Age Group 55	0.27 ± 0.11	<0.0054002	12
Age Group 70	0.19 ± 0.10	<0.024959	4
Year 12 (age adjusted)	0.47 ± 0.11	<0.0000076	7
Gender, BMI, smoker, drinker			
Age Group 40	0.39 ± 0.12	<0.0002241	15
Age Group 55	0.26 ± 0.11	<0.0069485	12
Age Group 70	0.18 ± 0.11	<0.0324463	4
Year 12 (age adjusted)	0.46 ± 0.11	<0.0000110	7

**Table 5:** Demographics

Variable	Age Group 40	Age Group 55	Age Group 70	Year 12 From Study Entry^A^
Mean Age (SD, N)	42.5 (3.3, 795)	55.3 (1.0, 795)	68.2 (3.2, 795)	52.5 (5.7, 781)
% Female (N)	55.6 (795)	55.6 (795)	55.6 (795)	55.4 (781)
Mean Height (SD, N)	65.2 (3.8, 792)	65.0 (3.7, 792)	64.3 (3.7, 792)	65.2 (3.7, 778)
Mean Weight (SD, N)	155.4 (29.4, 795)	159.4 (29.9, 795)	160.2 (31.3, 795)	158.6 (30.5, 765)
Mean BMI (SD, N)	25.6 (3.7, 792)	26.4 (3.9, 792)	27.1 (4.4, 792)	26.1 (4.0, 762)
Mean SBP (SD, N)	125.8 (16.2, 795)	131.7 (18.5, 795)	139.9 (20.6, 795)	130.9 (18.2, 768)
% Smoke (N)	54.1 (777)	36.6 (795)	17.2 (793)	40.3 (767)
% Drink (N)	76.2 (740)	71.0 (747)	58.3 (775)	69.2 (768)

^A^Visit 7 for Cohort 1 and visit 3 for Cohort 2.
